# Comparative Stability and Quality Assessment of Powder–Liquid Double-Chamber Bag Versus Traditional Meropenem Infusions: Implications for Critical Care and Individualized Dosing

**DOI:** 10.3390/pharmaceutics18030382

**Published:** 2026-03-20

**Authors:** Xiaokai Ren, Xiao Li, Liting Zhang, Xiaofei Zhao, Lei Zhang, Zhanjun Dong

**Affiliations:** 1 School of Pharmacy, Hebei Medical University, Shijiazhuang 050017, China; 23034101929@stu.hebmu.edu.cn; 2 Department of Pharmacy, Hebei General Hospital, Shijiazhuang 050051, China; zlt200100@163.com (L.Z.); zxf13731157412@163.com (X.Z.); m13383114065@163.com (L.Z.); 3 Hebei Key Laboratory of Clinical Pharmacy, Hebei General Hospital, Hebei Medical University, Shijiazhuang 050051, China

**Keywords:** meropenem, stability, finished infusions, insoluble particles, powder liquid double-chamber bags

## Abstract

**Background**: Maintaining therapeutic meropenem plasma concentrations requires prolonged infusion, but stability concerns exist between preparation and administration. This study compared the stability and operability of ready-to-use powder–liquid double-chamber bag (DCB) infusions versus traditional powder-for-injection (PFI) meropenem under clinical conditions. **Methods**: Infusions at clinically relevant concentrations were stored at 2–8 °C, 25 ± 5 °C, and 40 ± 2 °C for 12 h. Stability assessments included appearance, pH, osmolality, insoluble particle count, meropenem content (HPLC), and impurity A level. **Results**: DCBs demonstrated superior content uniformity, significantly fewer insoluble particles (*p* < 0.05), and greater operational simplicity compared to PFI. Refrigeration maintained meropenem content > 95% and effectively suppressed impurity formation for up to 12 h. However, at both room temperature and elevated temperature, impurity A exceeded pharmacopoeial limits within 2 h, particularly at higher concentrations. An innovative bedside solvent volume adjustment method enabled DCBs to deliver high-concentration infusions, facilitating individualized critical care dosing. **Conclusions**: Compared with traditional powder injection formulations, the Meropenem powder–liquid dual-chamber bag offers more convenient operation under routine preparation conditions and poses a lower risk of contamination during the preparation process. Its stability is more sensitive to storage temperature, requiring strict adherence to refrigeration conditions. When stored under standardized conditions, the dual-chamber bag can better ensure drug efficacy stability and medication safety, making it particularly suitable for clinical emergency use and standardized workflow management.

## 1. Introduction

As a representative second-generation carbapenem antibiotic [1], meropenem exerts its antibacterial effect by inhibiting bacterial cell wall mucopeptide synthetases (penicillin-binding proteins) and does not require co-administration of a dehydropeptidase inhibitor [2]. It exhibits potent efficacy against a wide range of aerobic and anaerobic Gram-positive and Gram-negative bacteria, including multidrug-resistant organisms and Pseudomonas aeruginosa. Clinical guidelines recommend meropenem for both empiric and targeted treatment of community-acquired severe pneumonia and severe abdominal infections. It is also recognized by the World Health Organization as a key agent for serious infections and is frequently selected as the first-line option for “step-down therapy” in intensive care unit (ICU) patients, particularly for high-risk infections such as nosocomial pneumonia and sepsis [3,4,5,6].

Amidst the growing challenge of bacterial resistance, meropenem remains indispensable in clinical anti-infective therapy due to its inherent activity against resistant pathogens. As a time-dependent antibiotic, its efficacy correlates positively with the time its plasma concentration exceeds the minimum inhibitory concentration (T > MIC). Prolonged infusions (e.g., over 3 h) can improve clinical response rates [7,8]. However, this is constrained by its solution stability (reported as 4–8 h) [9,10], imposing stricter timeliness requirements for the use of its finished infusions.

According to the Guidelines for the Construction and Management of Pharmacy Intravenous Admixture Services (PIVAS) [3], which aligns with international standards such as the United States Pharmacopeia (USP) General Chapter <797> for sterile compounding [11], antibiotics should ideally be compounded in PIVAS to mitigate risks such as contamination [12]. However, significant delays exist between the compounding and clinical administration of both long-term and on-demand prescription infusions, which can lead to meropenem degradation during storage and transport [13].

Meropenem and sodium chloride injection (powder–liquid dual-chamber bag) represents an innovative dosage form enabling immediate bedside preparation and administration. This offers advantages for urgent scenarios like emergency and critical care management of severe infections. Nonetheless, its domestic availability is recent, clinical use remains limited, and there is currently a lack of systematic comparative studies, both domestically and internationally, on the stability of this formulation versus traditional powder-for-injection finished infusions.

This study is the first to comprehensively evaluate, across multiple dimensions—including appearance, pH, active ingredient content, impurity profile, osmotic pressure, and insoluble particles—the stability differences between traditionally packaged meropenem finished infusions (original research and generic powder injections) and ready-to-use dual-chamber bag meropenem finished infusions under refrigeration (2–8 °C), room temperature (25 ± 5 °C), and elevated temperature (40 ± 2 °C) conditions. It aims to investigate the pharmaceutical innovation and value of the new packaging, provide a reference for rational clinical drug use, propose optimized usage scenarios and infusion time limits for different formulations based on stability data, and offer important evidence to support the optimization of carbapenem application guidelines and promote the evidence-based adoption of new formulations.

## 2. Materials and Methods

### 2.1. Instrumentation and Reagents

#### 2.1.1. Instrumentation

The following instruments were used in this study: ACQUITY H-Class ultra-performance liquid chromatograph (UPLC) (Waters Corporation, Milford, MA, USA); AB204-S electronic balance (METTLER TOLEDO, Greifensee, Switzerland; sensitivity: 0.1 mg); FE20 laboratory pH meter (Mettler-Toledo Instruments Co. Ltd., Shanghai, China); GWF-8JD particle analyzer (Tianjin Tianhe Analytical Instrument Co., Ltd., Tianjin, China); SMC 30C-1 osmotic pressure molar concentration meter (Tianjin Tianhe Analytical Instrument Co., Ltd., Tianjin, China); TGL-16 high-speed centrifuge (Shandong Baiou Medical Technology Co., Ltd., Qufu, China); and KQ3200E medical ultrasonic cleaner (Kunshan Ultrasonic Instrument Co., Ltd., Kunshan, China).

#### 2.1.2. Reagents

The reagents included meropenem/sodium chloride injection (Hunan Kelun Pharmaceutical Co., Ltd. Yueyang, China; 0.5 g, batch: L24100201A; 1.0 g, batch: L25020102SA); injectable meropenem (Sumitomo Pharma, Osaka, Japan; 0.5 g, batch: 2681C); meropenem for injection (Peking University Pharmaceutical Co., Ltd., Beijing, China; 0.5 g, batch: 240321); sodium chloride injection (Shijiazhuang Siyao Co., Ltd., Shijiazhuang, China; 100 mL, batch: 241043701); phosphoric acid (Tianjin Yongda Chemical Reagent Co., Ltd., Tianjin, China; batch: 20231017); triethylamine (Tianjin Obokai Chemical Co., Ltd., Tianjin, China; batch: 2024.7.20); meropenem standard substance (China Institute for Food and Drug Control, Beijing, China; batch: 130506-202305); meropenem impurity A standard (CATO Research Chemicals Inc., Charlotte, NC, USA; batch: 1053703-36-8); chromatographic-grade acetonitrile (Fisher Chemical, Thermo Fisher Scientific Inc., Waltham, MA, USA); and purified water (Hangzhou Wahaha Group Co., Ltd., Hangzhou, China).

### 2.2. Chromatographic Conditions

Chromatographic analysis was conducted using a WondaSil C18 Superb column (5 μm, 4.6 mm × 150 mm). The mobile phase consisted of a 0.1% triethylamine solution (prepared by dissolving 1.0 mL of triethylamine in 900 mL of water, adjusting the pH to 5.0 ± 0.1 with phosphoric acid, and diluting to 1000 mL) and acetonitrile at a ratio of 93.5:6.5 (*v*/*v*). The flow rate was 1.0 mL·min^−1^, the column temperature was set at 30 °C, the injection volume was 10 μL, and the injection chamber was maintained at 10 °C. Detection was performed at 220 nm.

According to the Chinese Pharmacopoeia, both meropenem and its impurities exhibit strong ultraviolet absorption at 220 nm. The chromatographic method employs an octadecylsilane-bonded silica gel stationary phase. In this study, an octylsilane-bonded silica gel column was used to replicate the pharmacopoeia conditions. Satisfactory separation was achieved between the meropenem ring-opening compound and the meropenem peak, meeting all relevant standards.

The impurity limit was defined as follows: The chromatogram retention time was recorded up to 3.5 times that of the principal component peak. If impurity peaks were observed in the test solution chromatogram, the maximum impurity peak area, both before and after the main peak, must not exceed 0.5% of the main peak area in the control solution. The sum of all individual impurity peak areas must not exceed 1.5% of the main peak area in the control. Peaks with areas less than 0.1 times the main peak area of the control solution were disregarded.

### 2.3. Analytical Indicators

Properties: The infusion should be a colorless to light yellow, clear liquid, allowing visual assessment of stability.

Solution clarity and color: The solution must remain clear and colorless; turbidity should not exceed that of Standard Turbidity Solution No. 1. The color should not be deeper than the Yellow or Yellow-Green 6 Standard Color Solution.

Content: Carbapenems are atypical β-lactam antibiotics, and their solutions are typically unstable and prone to hydrolysis, resulting in reduced content. The stability of the infusion product was evaluated by monitoring content changes, which should remain within 90–110% in accordance with Chinese Pharmacopoeia requirements.

Related substances: Based on the 2020 edition of the Chinese Pharmacopoeia and preliminary findings indicating a significant increase in impurity A, this study focused on monitoring changes in impurity A.

### 2.4. Preparation of Solutions

Analytical solutions, including reference standards, quality controls, system suitability solutions, and simulated clinical infusions, were prepared to evaluate the stability and content of meropenem.

#### 2.4.1. Preparation of Standards, Control Solutions and Test Samples

All analytical weighing was performed using a calibrated five-decimal balance (sensitivity 0.01 mg), with volumetric measurements conducted using amber Class A glassware to mitigate the inherent instability of meropenem.

A 5 mg·mL^−1^ meropenem standard stock solution was prepared by accurately weighing approximately 50 mg of the reference standard, corrected for purity and water content, and dissolving it in deionized water. This stock was quantitatively diluted to 1 mg·mL^−1^ to obtain the working standard solution. For impurity analysis, a 0.1 mg·mL^−1^ impurity A standard solution was prepared by dissolving 1 mg of the reference standard in a 10 mL amber volumetric flask.

To ensure sampling homogeneity, the entire contents of the meropenem dosage forms, including powder injections and dual-chamber bags, were thoroughly homogenized prior to weighing a 10 mg aliquot. The test solution (1 mg·mL^−1^) was prepared by dissolving the aliquot in water through 1 min of vortexing followed by 5 min of isothermal sonication (100 W, 25 ± 2 °C) to prevent thermal degradation. A 25 μg·mL^−1^ control solution was subsequently prepared by diluting 2.5 mL of the test solution to 100 mL, representing the 0.5% impurity limit in accordance with the related substances specifications of the Chinese Pharmacopoeia (2020 Edition).

All prepared solutions were filtered through 0.22 μm polytetrafluoroethylene (PTFE) membranes and analyzed within 30 min of constitution to minimize hydrolytic degradation.

#### 2.4.2. Preparation of Finished Infusion Solution

To ensure operational consistency and accurately simulate real-world clinical workflows, all infusion preparations described below were uniformly performed by certified pharmacists in accordance with standard compounding protocols.

(1) Traditional configuration group:

Ward-based preparation: Infusions were prepared by adding 0.5 g, 1.0 g, or 2.0 g of meropenem for injection to 100 mL of 0.9% sodium chloride injection, resulting in final concentrations of 5 mg mL^−1^, 10 mg mL^−1^, and 20 mg mL^−1^, respectively.

PIVAS-based preparation: At the PIVAS horizontal laminar flow bench, infusions were compounded using the identical doses and solvent volumes, yielding the same final concentrations of 5 mg·mL^−1^, 10 mg·mL^−1^, and 20 mg·mL^−1^, respectively.

(2) Powder–liquid double-chamber bag (DCB) group:

DCB preparation: Meropenem/sodium chloride injections (0.5 g and 1.0 g) were activated by pressing to open the seal between chambers, thoroughly mixing the powder and solvent to achieve final concentrations of 5 mg·mL^−1^ and 10 mg·mL^−1^. For the 2.0 g group, two 1.0 g DCBs were used; after withdrawing 50 mL of sodium chloride from one bag, the powder was mixed with the remaining solvent, resulting in a final concentration of 20 mg mL^−1^.

Content determination: Aliquots of 20 mL (from the 5 mg·mL^−1^ group), 10 mL (from the 10 mg·mL^−1^ group), and 5 mL (from the 20 mg mL^−1^ group) were collected and diluted to 100 mL with water for quantitative analysis.

#### 2.4.3. System Adaptation Testing

**Figure 1:**
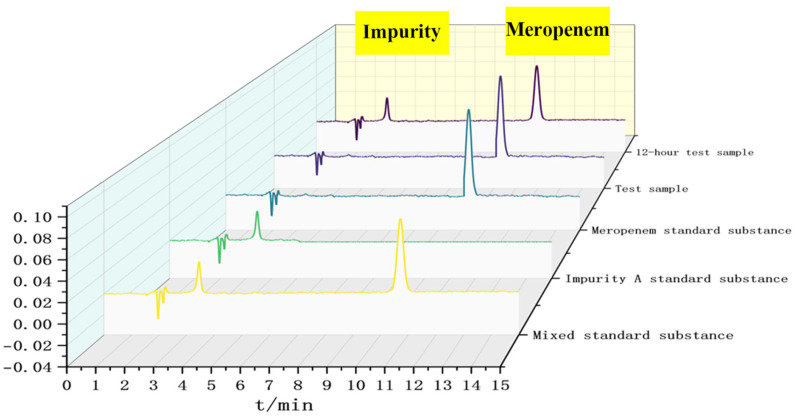
UPLC chromatograms of the test and standard samples.

The control and test solutions were analyzed using the chromatographic conditions described in Section 2.2, and chromatograms were recorded (Figure 1). Figure 1 presents a superimposed ultra-high-performance liquid chromatography (UHPLC) chromatogram of the meropenem reference standard, meropenem impurity A reference standard, and meropenem test samples (including both the traditional powder injection preparation group and the powder–liquid dual-chamber bag preparation group) under the chromatographic conditions specified in Section 2.2. The x-axis represents retention time (t/min, range 0–15 min), and the y-axis indicates the chromatographic peak response (in AU). This figure clearly illustrates the separation efficiency, retention time characteristics, and peak shape integrity of each component, providing direct visual validation for the subsequent methodological effectiveness of content determination and related substance analysis.

### 2.5. Method Validation for Content Determination

To ensure the analytical reliability and quantitative accuracy of the established HPLC method, comprehensive methodological validation was performed. The evaluated parameters encompassed the limit of detection (LOD), limit of quantification (LOQ), linearity, precision, repeatability, and analytical recovery.

#### 2.5.1. Limit of Detection and Limit of Quantification

A series of meropenem reference solutions at varying concentrations was prepared. The LOD was defined as the concentration corresponding to a signal-to-noise ratio (S/N) of 3, and the LOQ as the concentration at *S*/*N* = 10. The LOD and LOQ for meropenem were 14.52 μg·mL^−1^ and 41.58 μg·mL^−1^, respectively.

#### 2.5.2. Standard Curve Establishment

Precision was evaluated by transferring 1, 1.5, 2, 2.5, and 3 mL of meropenem standard solution into 20 mL volumetric flasks and diluting to volume with water to obtain solutions with concentrations ranging from 0.25 to 1.5 mg·mL^−1^. The peak area (Y) regressed against concentration (X), resulting in the equation Y = 1.776 × 10^−4^X − 14.78932, with a correlation coefficient (r) of 0.99996. The linear range was 0.25–1.5 mg·mL^−1^.

#### 2.5.3. Precision and Repeatability

Precision test: Six consecutive injections (10 μL each) of meropenem control solution (1 mg·mL^−1^) yielded a relative standard deviation (RSD) of 0.21% for peak area, indicating high instrument precision.

Repeatability test: Six parallel preparations of finished meropenem infusion were analyzed, with RSDs of peak area of 0.48%, 0.45%, and 0.54% (*n* = 6), demonstrating good method reproducibility.

#### 2.5.4. Recovery Experiments

Test solutions were prepared as described in Section 2.4.2. Precisely measured 5 mL aliquots were placed in 10 mL volumetric flasks, spiked with additional meropenem at concentrations corresponding to 80%, 100%, and 120% of the initial test solution, and diluted to volume. Samples were injected, and peak areas were recorded and referenced to the standard curve. Recovery was calculated as (measured added amount − original content)/amount added. The recoveries were 98.73%, 99.69%, and 98.11% for 80%, 100%, and 120% spiking, respectively, with RSDs of 0.54%, 0.71%, and 0.23%.

### 2.6. Infusion Stability Study

Each sample was labeled numerically, and detailed corresponding conditions are presented in Table 1. This study employed a systematic experimental design to evaluate the stability of Meropenem finished infusions, considering three key variables: storage temperature (Refrigeration: 2–8 °C; Room Temperature: 25 ± 5 °C; High Temperature: 40 ± 2 °C), drug specification (0.5 g, 1.0 g, 2.0 g), and preparation environment (hospital Ward vs. PIVAS). The formulations tested included the originator product (Meiping), a generic equivalent (Zhuojie), and an innovative powder–liquid dual-chamber bag. A clear and logical coding system was established to uniquely identify each test group. The sample code follows the structure: [Dosage]-[Formulation/Preparation Abbreviation]-[Temperature Abbreviation]. For instance, the code 10-MW-R decodes to a 1.0 g specification of the originator product (M), prepared in the Ward (W), and stored at Room Temperature (R). This structured design and coding ensure precise tracking and comparison of all experimental conditions.

The content and related substances were determined using the methods described in this study, while other quality indicators were assessed according to the General Rules of Volume IV of the People’s Republic of China Pharmacopoeia (2020 edition). Data were processed using SPSS 21.0 and are expressed as mean ± SD. Standard curves were recalibrated every 24 h, and system suitability was confirmed by retention time and peak shape for each batch.

For each combination of formulation, concentration, and storage condition, eight independent infusion solutions were prepared and analyzed at each time point (*n* = 8), with results expressed as mean ± SD. For the subsequent stability-related figures, the red dots in the plots represent the mean values of the eight replicate measurements at each time point, which intuitively reflect the central tendency of the experimental data.

## 3. Results

### 3.1. Appearance

**Figure 2:**
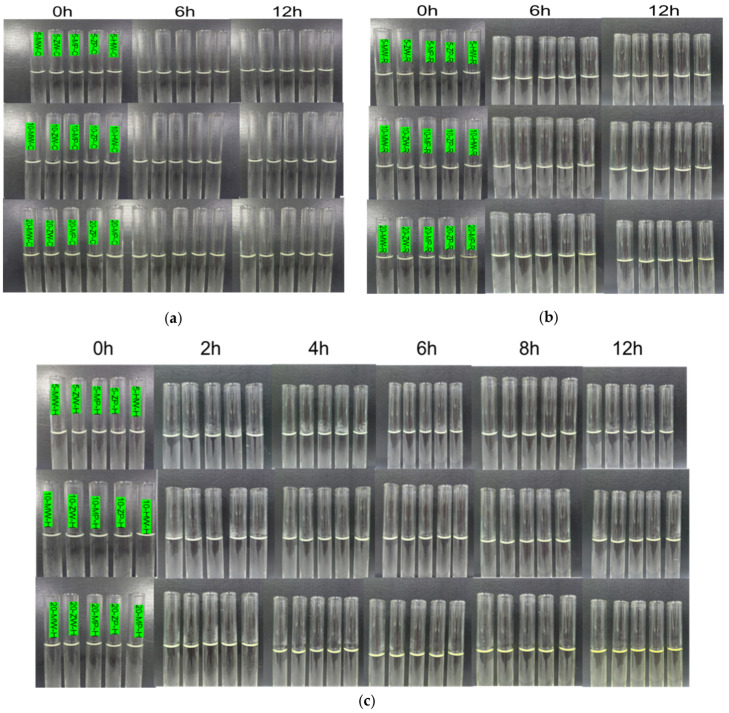
Changes in solution appearance over 0–12 h for each group: (**a**) refrigeration group; (**b**) room temperature group; (**c**) high temperature group.

Solution color: Storage temperature had a marked effect on color stability. At 40 ± 2 °C, discoloration was observed after 4 h, with greater changes at higher concentrations and longer storage times. At 25 ± 5 °C, color changes appeared only after 12 h. No color changes were detected within 12 h in the refrigeration group (2–8 °C). Across all preparation methods (traditional ward, PIVAS, DCB), similar trends were observed, with no significant differences (Figure 2).

Clarity and visible foreign bodies: Storage temperature did not affect solution clarity or the presence of visible particulates. No turbidity, precipitation, or visible foreign bodies were observed in any group. The three preparation groups showed consistent results under identical conditions (Figure 2).

### 3.2. pH

**Figure 3:**
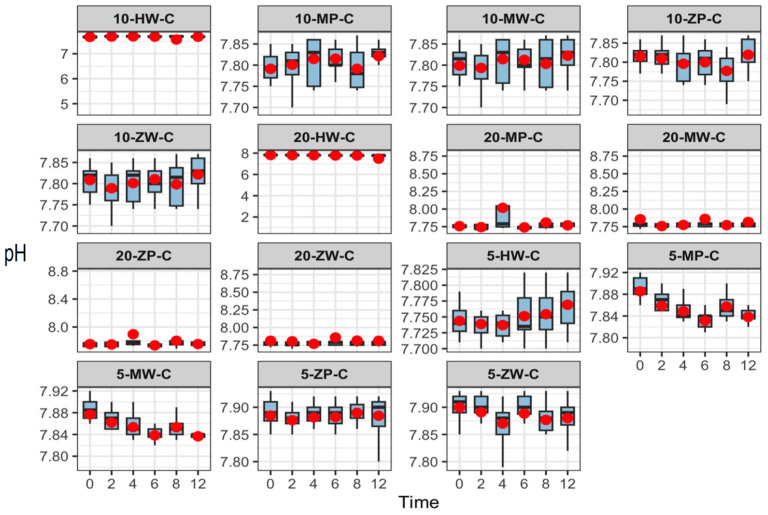
pH stability of meropenem finished infusions at various concentrations stored under refrigeration over 0–12 h. The red dots represent the mean values of eight independent replicate measurements (*n* = 8) at each time point.

**Figure 4:**
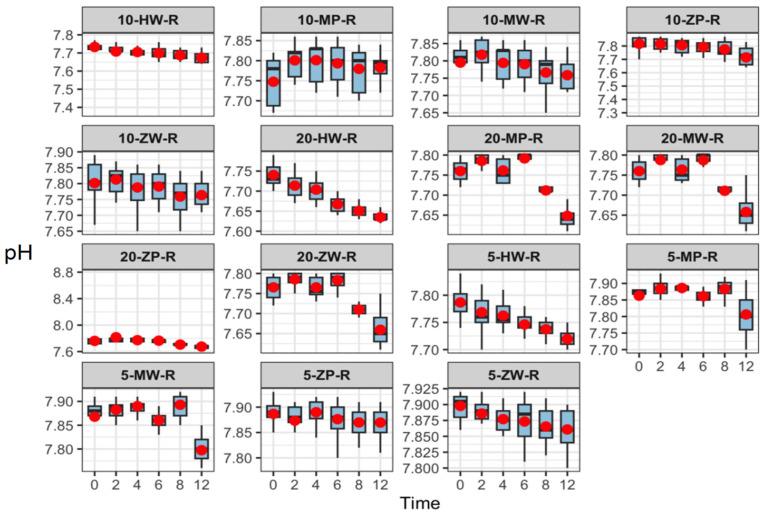
pH stability of meropenem finished infusions at various concentrations stored at room temperature over 0–12 h. Red dots are defined as in Figure 3.

**Figure 5:**
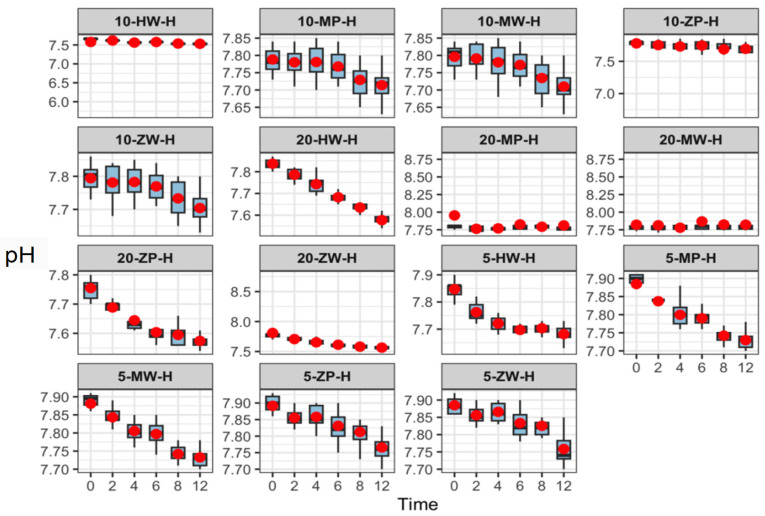
pH stability of meropenem finished infusions at various concentrations under high-temperature conditions over 0–12 h. Red dots are defined as in Figure 3.

Over 12 h, pH values across all groups ranged from 7.47 to 7.95. With increasing storage time, pH values decreased slightly in the room-temperature and high-temperature groups but remained stable under refrigeration. No significant differences were observed among parallel groups or across preparation methods at the same temperature and concentration. Results are presented in Figure 3, Figure 4 and Figure 5.

### 3.3. Insoluble Particles

Insoluble particle analysis was conducted according to Section 4 of the Chinese Pharmacopoeia (2020 edition). Data were analyzed using Minitab^®^ 21 (Minitab Inc., State College, PA, USA). Normality was assessed using the Anderson–Darling test and homogeneity of variance with Bartlett’s test. For data with homogeneous variances, one-way ANOVA followed by Tukey’s post hoc test was performed; for heterogeneous variances, alternative post hoc tests were applied. And sort the averages of particulate matter count across different configurations in descending order. Label the largest average as “a”. Compare this average with each following one: Label those with no significant difference as “a” until a significantly different average is found, which is labeled “b”. Then take the “b”-labeled average as the benchmark, compare it with all larger averages above, and label non-significant ones as “b” until a significantly different one is labeled “c”. Averages with the same letter are not significantly different, while those with different letters are.

At 0 h, significant differences were observed in the counts of insoluble particles sized 2 μm and 5 μm across different preparation environments. For 2 μm particles, a significant difference was observed among groups (F = 674.239, *p* < 0.001): Traditional ward group > traditional PIVAS group > DCB group. The same order was observed for 5 μm particles (F = 387.917, *p* < 0.001).

At 0 h, significant differences were also observed in 2 μm and 5 μm particle counts across different dose groups: 20 mg > 10 mg > 5 mg for both particle sizes (F = 47.865 and F = 20.329, respectively; both *p* < 0.001).

At 0 h, the concentrations of 10 μm and 25 μm insoluble particles in all groups and environments complied with the Pharmacopeia requirements (Table 2).

### 3.4. Osmotic Pressure

**Figure 6:**
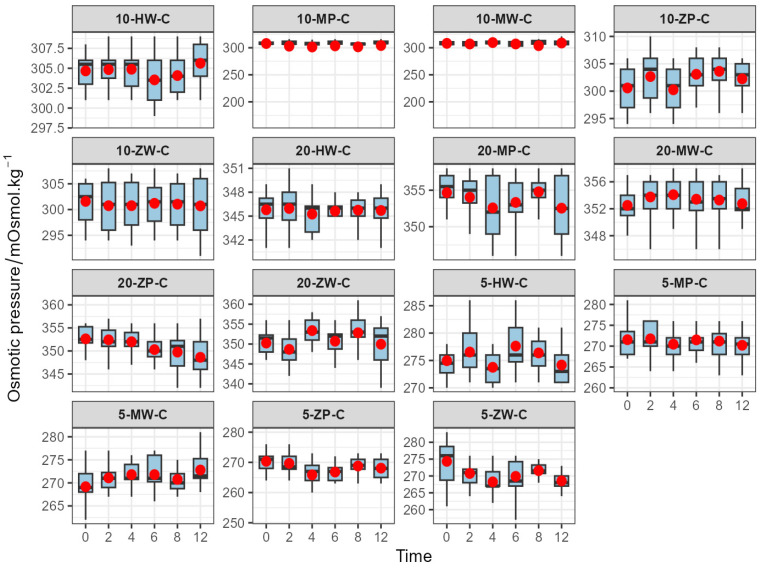
Line chart of Osmotic pressure changes in 5 mg/mL concentration groups of meropenem finished infusions under refrigeration over 0–12 h. Red dots are defined as in Figure 3.

**Figure 7:**
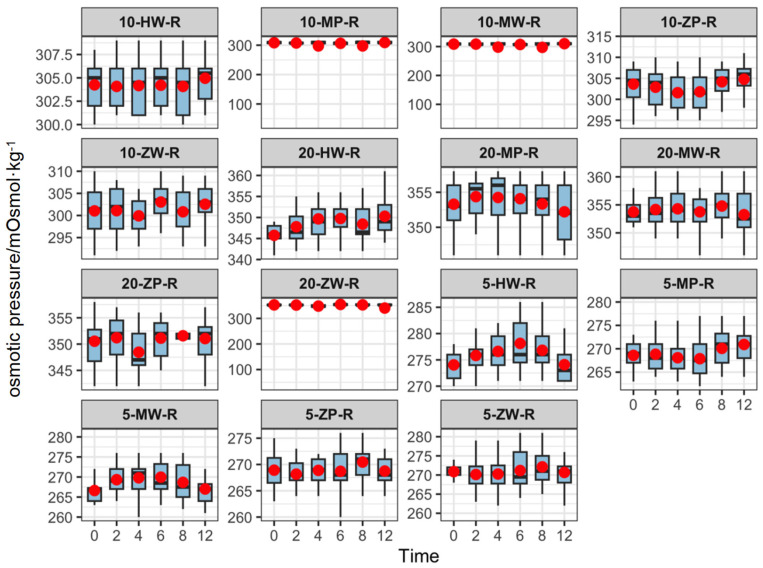
Line chart of Osmotic pressure changes in 10 mg/mL concentration groups of meropenem finished infusions under refrigeration over 0–12 h. Red dots are defined as in Figure 3.

**Figure 8:**
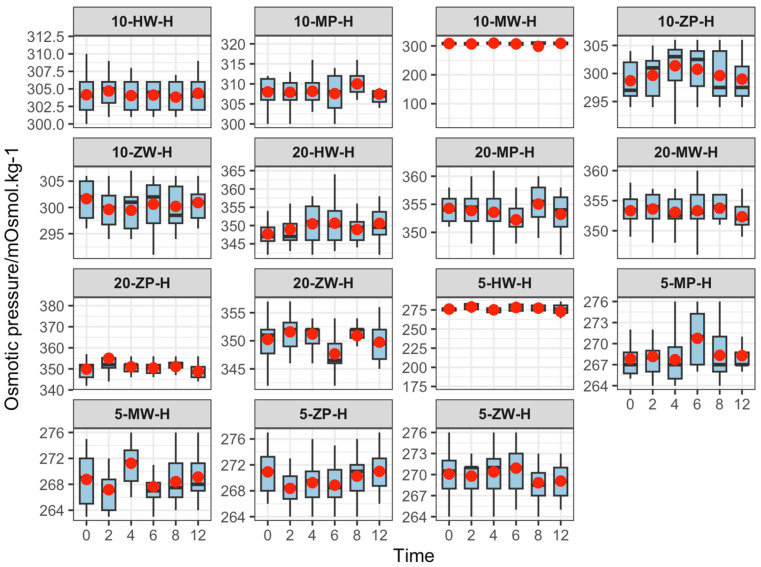
Line chart of Osmotic pressure changes in 20 mg/mL concentration groups of meropenem finished infusions under refrigeration over 0–12 h. Red dots are defined as in Figure 3.

The osmotic pressure of the finished products ranged from 265.88 to 355.04 mOsmol·kg^−1^, which is comparable to the physiological range of human blood (285–310 mOsmol·kg^−1^). Osmotic pressure remained stable over time at all temperatures, as shown in Figure 6, Figure 7 and Figure 8.

### 3.5. Changes in Meropenem Content and Impurity A

Table 3 summarizes the 0-h concentration, concentration range, and RSD of meropenem prepared by different methods and concentrations. The DCB group demonstrated higher mean concentrations (107.3%, 106.9%, and 108.3% of the theoretical value for 0.5 g/100 mL, 1.0 g/100 mL, and 2.0 g/100 mL, respectively) and narrower concentration ranges than the traditional group. The RSD was significantly lower in the DCB group (1.73%, 1.29%, and 1.40%) compared to the traditional group (3.33%, 3.58%, and 4.36%), indicating reduced drug loss and improved uniformity during preparation. In the traditional group, mean concentrations were generally lower than theoretical values, especially at high concentrations (e.g., the mean for 2.0 g/100 mL was 1975.1 mg/100 mL, representing a −1.2% deviation from the theoretical value of 2000 mg/100 mL), probably due to physical adsorption or volatilization during preparation.

**Figure 9:**
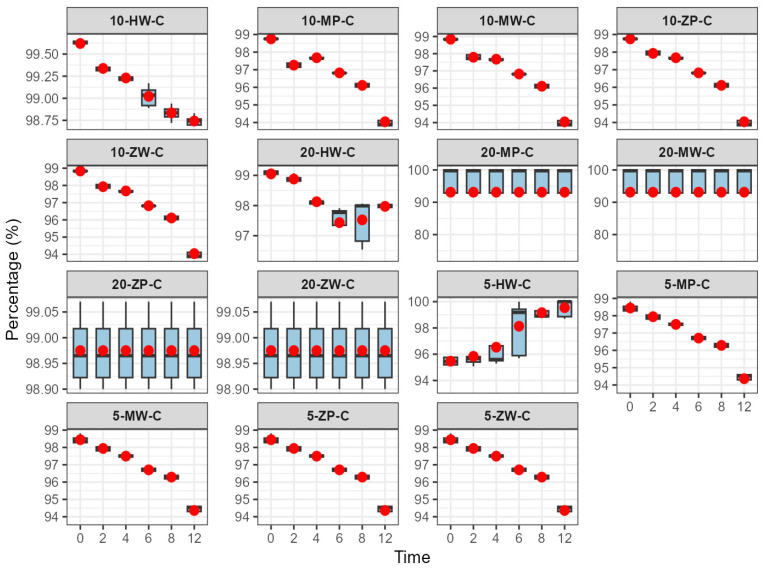
Content change trend of meropenem infusion under refrigerated conditions within 0–12 h. Red dots are defined as in Figure 3.

**Figure 10:**
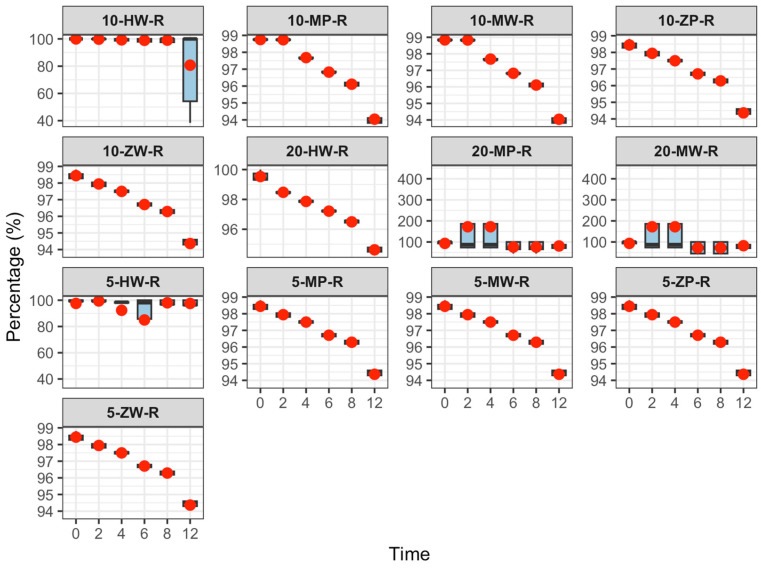
Content change trend of meropenem infusion under room temperature conditions within 0–12 h. Red dots are defined as in Figure 3.

**Figure 11:**
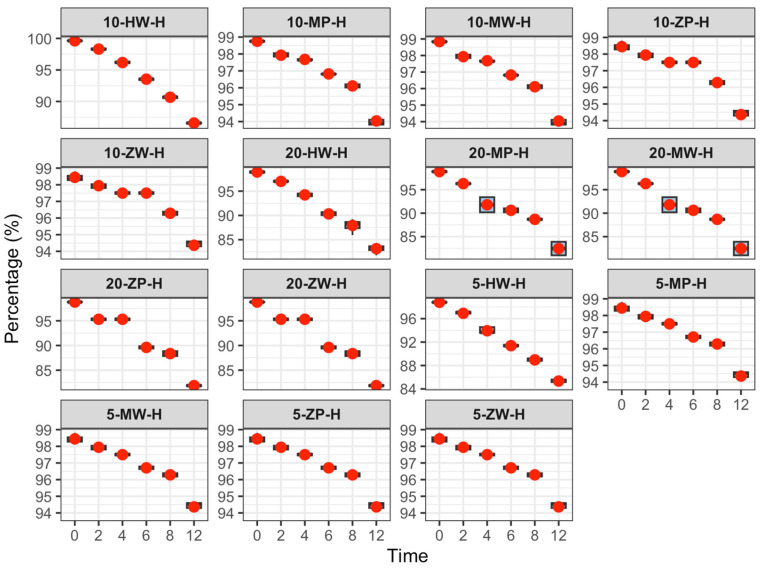
Content change trend of meropenem infusion under high temperature conditions within 0–12 h. Red dots are defined as in Figure 3.

The content change results are shown in Table 4 and Figure 9, Figure 10 and Figure 11:

Refrigeration (2–8 °C): No significant change in meropenem content was observed within 12 h, and 12-h values remained within the Pharmacopoeia standard (90–110%).

Room temperature (25 ± 5 °C): Meropenem content decreased slightly over 12 h. The high-concentration DCB group failed to meet the minimum Pharmacopoeia requirement at 12 h, while other groups remained compliant.

High temperature (40 ± 2 °C): Elevated temperature significantly accelerated degradation, and the 12-h content decrease was positively correlated with concentration. In the low-concentration DCB group, content did not meet Pharmacopoeia requirements after 8 h; in other groups, this occurred after 6 h. In the medium-concentration group, all preparations failed to meet requirements at 6 h, and in the high-concentration group, all failed at 4 h. These results indicate that meropenem is susceptible to hydrolysis or oxidation at high temperatures.

One-way ANOVA revealed no significant difference in content reduction among concentration groups under refrigeration (*p* > 0.05). At room temperature, content reduction in the low-concentration group was significantly lower than that in the medium- and high-concentration groups (*p* < 0.05). Under high temperature, content reduction followed the order high > medium > low concentration, with significant differences between groups (*p* < 0.05).

**Figure 12:**
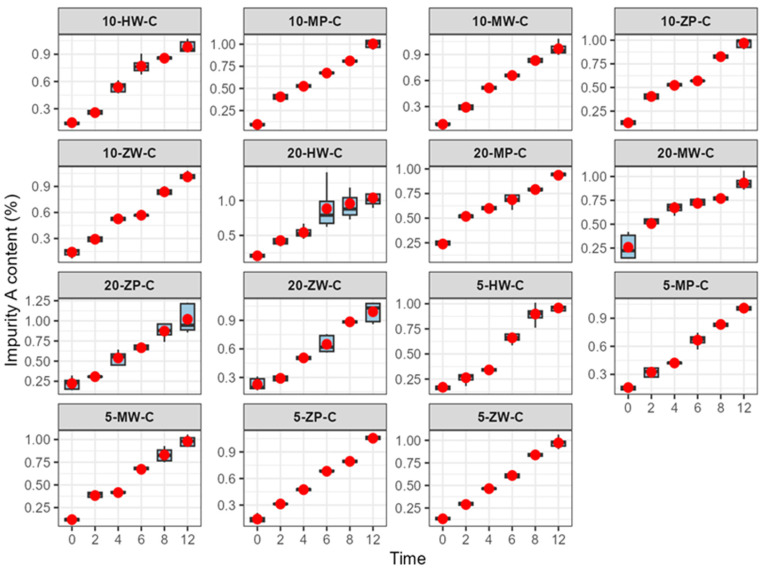
Content change trend of Impurity A in meropenem infusion under refrigerated conditions within 0–12 h. Red dots are defined as in Figure 3.

**Figure 13:**
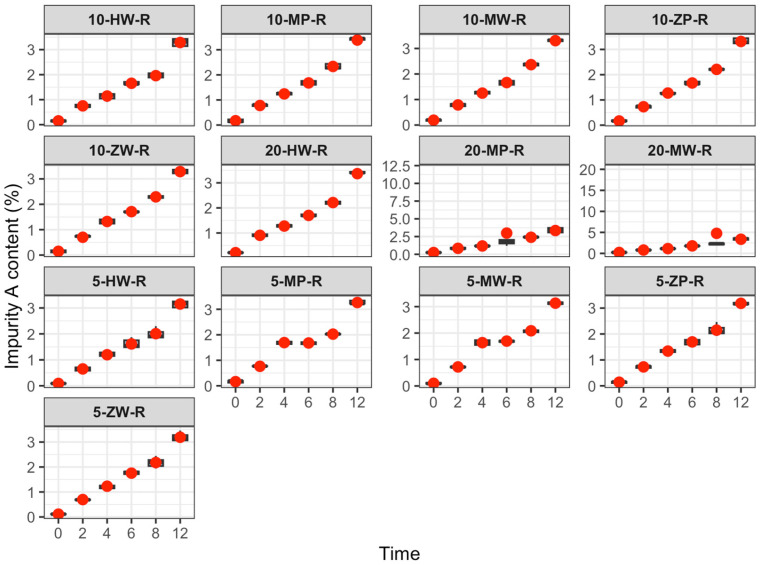
Content change trend of Impurity A in meropenem infusion under room temperature conditions within 0–12 h. Red dots are defined as in Figure 3.

**Figure 14:**
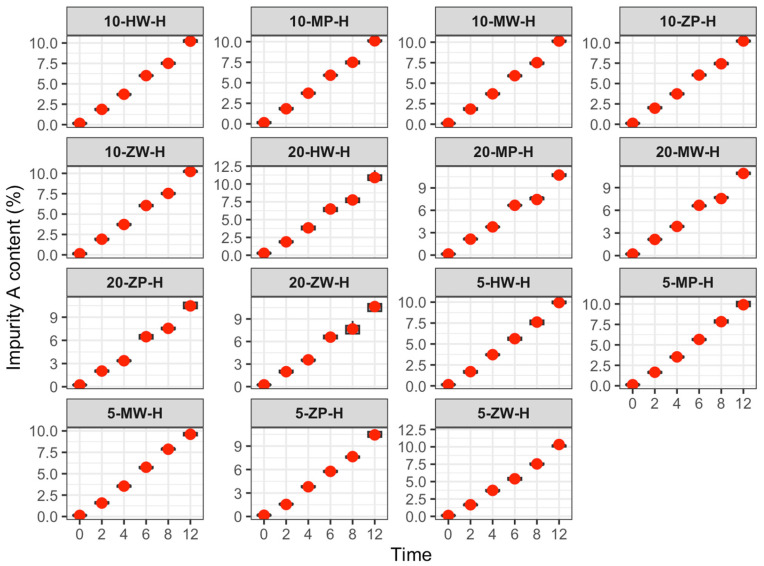
Content change trend of Impurity A in meropenem infusion under high temperature conditions within 0–12 h. Red dots are defined as in Figure 3.

Impurity changes are shown in Table 5 and Figure 12, Figure 13 and Figure 14:

Refrigeration (2–8 °C): Impurity A exceeded the Chinese Pharmacopoeia limit (<0.5%) as follows: Low-concentration group after 6 h; medium-concentration group (traditional group after 4 h, DCB groups after 6 h); high-concentration group (traditional group after 2 h, PIVAS and DCB groups after 4 h).

Room temperature (25 ± 5 °C): At 2 h, impurity A exceeded the Pharmacopoeia limit (<0.5%) in all groups.

High temperature (40 ± 2 °C): At 2 h, impurity A exceeded the Pharmacopoeia limit (<0.5%) in all groups.

## 4. Discussion

### 4.1. Comparative Analysis of Meropenem Formulations and Dosing Strategies

Although the physicochemical instability of meropenem is well recognized, prior investigations have primarily relied on simulated laboratory conditions or focused on single compounding methods. Table 6 contextualizes the current findings against recent literature to highlight these methodological distinctions.

Rather than restricting the evaluation to basic active ingredient recovery, this study introduces a comprehensive assessment aligned with the Chinese Pharmacopoeia and ICH guidelines. It systematically compares real-world compounding environments (PIVAS versus ward-based preparation) and benchmarks the powder–liquid DCB against traditional configuration methods. By tracking specific degradation products and insoluble particulates alongside standard assays, this approach generates clinically translatable data for optimizing meropenem administration.

Beyond compounding methods, formulation variability between generic and original products remains a critical factor. While previous European studies have reported similar in vitro efficacy between generic and original meropenem, documented discrepancies in stability, degradation, solubility, and dissolution rates can still directly influence clinical outcomes.

This study is the first in China to systematically compare two generic meropenem formulations with the original meropenem product. The results indicated no significant differences in the active pharmaceutical ingredient content or in vitro antibacterial activity. Nevertheless, discrepancies were observed in certain characteristics—specifically, ring-opening metabolites, residual solvents, insoluble particles, and dissolution time—between the original and generic meropenem formulations. A comprehensive comparison was performed to assess the stability of powder/liquid DCB meropenem injection (generic drug) and traditional meropenem injection (both original and generic drugs) under various temperatures and preparation conditions. The key quality indicators evaluated were appearance, pH, osmotic pressure, insoluble particles, content, and related substances.

Dosage selection was based on established clinical research data. For pneumonia, urinary tract infection, and gynecological infection, the recommended dose is 0.5 g every 8 h. For hospital-acquired pneumonia, peritonitis, neutropenia with confirmed infection, and sepsis, the recommended dose is 1 g every 8 h. For meningitis, 2 g every 8 h is recommended. Therefore, three dose levels—0.5 g, 1.0 g, and 2.0 g—were included in this study to reflect clinical practice.

Regarding dosage forms, traditional powder injections allow flexible adjustment based on actual treatment needs, owing to their operational flexibility. Although the powder–liquid DCB allows for aseptic, ready-to-use administration, only two fixed specifications are currently available: 0.5 g/50 mL and 1.0 g/100 mL. These fixed doses may not meet the needs of certain specialized clinical scenarios. In critical care settings, such as the ICU, achieving target PK/PD parameters often requires a high-dose regimen of 2 g/100 mL, which presents challenges for the use of powder/liquid DCBs.

To address this limitation, an innovative volume control technique was developed to pre-extract 50 mL of solvent from the liquid DCB, followed by thorough mixing to achieve a high-concentration infusion of 2 g/100 mL. Clinical adaptability analysis showed that this method preserves the benefits of DCBs—ready-to-use administration and airtight configuration—while allowing flexible bedside dose adjustment. This approach enables precise alignment with individualized treatment needs in critically ill patients and offers a novel technical strategy for using DCB preparations in the management of septic shock and other critical conditions requiring personalized therapy.

### 4.2. Impact of Temperature on Infusion Stability

This study evaluated the stability of meropenem infusion solutions under three temperature conditions: cold storage (2–8 °C), room temperature (25 ± 5 °C), and high temperature (40 ± 2 °C). In clinical practice, meropenem is often administered in critical care settings, such as ICUs, where continuous infusion and the prolonged proximity of tubing to the patient’s body can increase the temperature of both the tubing and its contents. The Sanford Antimicrobial Therapy Guidelines [16] note that body heat can raise the temperature of antibiotics delivered through portable infusion pumps to nearly 37 °C. Carbapenems, including meropenem, are notably unstable under these conditions [17], often necessitating cooling pads for infusion pumps or frequent replacement of infusion bags and tubing. Investigating meropenem stability at high temperatures (40 ± 2 °C), therefore, provides valuable clinical guidance.

### 4.3. Comprehensive Evaluation of Meropenem Infusion Quality and Stability

#### 4.3.1. Appearance

The appearance of the infusion solution serves as a direct visual indicator of stability. In this study, meropenem infusions exhibited color changes after 4 h at high temperatures, with more pronounced darkening at higher concentrations. In contrast, no significant color changes were detected within 0–12 h under refrigerated or room temperature conditions, regardless of configuration.

#### 4.3.2. pH

The β-lactam ring of meropenem is most stable in near-neutral solutions [18], while alkaline conditions may lead to the formation of degradation products such as ring-openers and decarboxylated compounds [19]. In this study, the pH of finished meropenem infusions remained stable over 0–12 h across all temperatures and configurations.

#### 4.3.3. Osmotic Pressure

Osmolality depends on solute particle concentration and directly correlates with solute content. Throughout the 0–12 h period, osmotic concentrations ranged from 265.88 to 355.04 mOsmol·kg^−1^, with no significant fluctuations. Osmotic pressure increased with drug concentration but remained within the physiological range of human blood (285–310 mOsmol·kg^−1^). All experimental groups maintained stable osmotic pressure within 12 h.

#### 4.3.4. Ingredient Content

Regarding drug content uniformity, powder and liquid DCB infusions exhibited a higher mean concentration, a narrower concentration range, and a significantly lower RSD compared to traditional preparations. The mean concentration of traditional powder injections was often below the theoretical value, due to physical adsorption, transfer loss, or volatilization. The powder–liquid DCB preparation offers ready-to-use conditions, preserving both drug concentration and dosing accuracy, thereby minimizing configuration losses and enhancing clinical effectiveness.

Under cold storage (2–8 °C), meropenem content remained stable, with an average decrease of less than 1.5% over 12 h. Environmental factors and drug concentration had minimal impact on component degradation, and meropenem content at 12 h satisfied Chinese Pharmacopoeia standards. At room temperature (25 ± 5 °C), meropenem content decreased linearly with time, and higher concentrations experienced greater reductions. At 12 h, the high-concentration powder and liquid DCB group exhibited a 12.3% decrease, failing to meet the Pharmacopoeia requirements, whereas the other groups remained within standards. Elevated temperatures (40 ± 2 °C) substantially accelerated meropenem degradation. At 12 h, the reduction in content positively correlated with concentration, with higher concentrations declining more rapidly. In the low-concentration group, meropenem content dropped below the Pharmacopoeia minimum between 6 and 8 h; in the medium-concentration group, this occurred at 6 h; and by 4 h, all groups failed to meet Pharmacopoeia requirements.

These findings indicate that meropenem infusion stability is affected by preparation method, storage conditions, and concentration. Powder and liquid DCBs enhance concentration uniformity, and it is recommended that qualified medical institutions prioritize this option. Refrigerated storage is advised to extend the efficacy of meropenem infusion products, while immediate use is necessary at high temperatures to prevent degradation. Higher concentrations show greater content loss over time, necessitating stringent temperature and time controls for medium and high concentrations (≥10 mg/100 mL) to ensure safety and efficacy.

In the establishment of impurity limits, China’s drug evaluation system primarily references international standards, such as those of the International Council for Harmonization of Technical Requirements for Pharmaceuticals for Human Use (ICH) [20,21]. Traditional drug evaluation has focused on quality and safety, often overlooking individual variability and actual clinical needs. Drug impurities significantly affect safety, quality, and efficacy; thus, effective impurity control is essential to protect patients. Currently, domestic impurity oversight is concentrated in the research, development, and registration phases, with limited monitoring during actual drug use. The Technical Requirements for Consistency Evaluation of Quality and Efficacy of Generic Chemical Injections (No. 2, 2020) do not specify detailed requirements for the “stability of finished infusion products,” nor are there clear guidelines on impurity growth limits in meropenem infusion products over time.

In this study, following the Chinese Pharmacopoeia standard for meropenem (maximum single impurity < 0.5%) [22], the variation in impurity A was investigated over 0–12 h across different environments. Results showed that under room temperature (25 ± 5 °C) and high-temperature (40 ± 2 °C) conditions, impurity A exceeded the maximum allowable limit within 2 h, regardless of the specific environment. Furthermore, the growth rate of impurity A was positively correlated with concentration. Under cold storage (2–8 °C), impurity A exceeded the limit at 6 h in both the low-concentration group and the medium-concentration DCB group; at 4 h in the medium-concentration traditional configuration group, the high-concentration traditional configuration group, and the high-concentration DCB group; and at 2 h in the high-concentration traditional configuration original group.

Global labeling for meropenem recommends using infusions within 6 h at room temperature. However, this study found that impurity A exceeded 1.3–1.8 times the Pharmacopoeia limit after 2 h and reached 3.2–3.5 times the limit after 6 h at room temperature. Under high-temperature conditions, impurity A exceeded 3.1–4.3 times the limit within 2 h. Any substance surpassing the Pharmacopoeia limit is deemed noncompliant. Insufficient quality control after reconstitution may result in impurity A exceeding the standard within the usage timeframe specified in the product labeling.

Studies have shown that meropenem is prone to non-enzymatic degradation both in vivo and in vitro. The core β-lactam ring, essential for its antibacterial activity, is susceptible to hydrolysis, forming a ring-opened metabolite. This process occurs not only in vitro, such as in prepared solutions, but also in vivo, where the ring-opened metabolite is the major product of meropenem metabolism by renal dehydropeptidase-I and completely lacks antibacterial activity [23]. The opening of the β-lactam ring exposes new functional groups and reactive sites, potentially allowing the metabolite to act as a “hapten” that irreversibly binds to serum proteins (e.g., albumin) to form a complete antigen. This can then trigger a specific immune response and, in severe cases, allergic reactions—a mechanism analogous to that of penicillin and other β-lactam drugs, where degradation products conjugate with proteins to induce allergies.

Clinical observations indicate significant individual variability in the ratio of the ring-opened metabolite to total meropenem concentrations in critically ill patients. In patients with renal impairment, the half-life of the ring-opened metabolite is approximately 35 h, leading to substantial accumulation and theoretically increasing the risk of protein conjugation and subsequent immune reactions. Furthermore, special populations such as critically ill patients are more sensitive to drug impurities, which may initiate or exacerbate organ dysfunction, elevate the risk of allergic reactions, and in severe cases, be life-threatening.

Therefore, greater clinical attention should be paid to the potential risks posed by the ring-opened metabolite. It is recommended that meropenem finished infusions be prepared and administered immediately with strict temperature control. There is also a call for regulatory authorities to improve impurity evaluation standards to better ensure medication safety in the future [23,24,25].

#### 4.3.5. Insoluble Particles

Insoluble particles are foreign substances with diameters less than 50 μm that cannot be metabolized by the body. Excluding air bubbles, these particles may be introduced during the production or administration of injections and serve as a key indicator for distinguishing between traditional dispensing methods and powder–liquid DCB methods. According to the National Annual Report on Adverse Drug Reaction Monitoring (2024), 57.2% of adverse drug reaction or event reports involved injections, of which 91% were intravenous injections [26]. Literature indicates that excessive insoluble particulate matter is a primary cause of infusion-related adverse reactions [27]. Once introduced into the bloodstream, these particles may lodge in small vessels, causing local embolic injury, tissue necrosis, and microvascular occlusion. Repeated exposure may also result in inflammation and granuloma formation [28].

This study assessed insoluble particles larger than 10 μm and 25 μm, as required by the Chinese Pharmacopoeia, and additionally quantified particles exceeding 2 μm and 5 μm. While the counts for Pharmacopoeia-defined particle sizes were within normal ranges, significant differences were observed in the numbers of 2 μm and 5 μm particles among infusion formulations prepared in different environments. All finished infusion products were analyzed by a single professional pharmacist across various environments, manufacturers, and concentrations, minimizing measurement errors and ensuring data accuracy.

Multiple factors during drug compatibility—including environment, syringe type, operator technique, drug opening method, disinfection practices, and the number of punctures—collectively increase the particle count in the final solution [19].

Infusions prepared using the traditional PIVAS configuration contained significantly fewer insoluble 2 μm and 5 μm particles than those prepared in the ward. This difference is attributable to the use of a Class 100 biological safety cabinet in PIVAS, which effectively reduces environmental contamination. The PIVAS system not only enhances drug utilization and infusion safety but also reduces occupational risks for healthcare staff [26]. Moreover, compared with both traditional methods, the powder–liquid DCB approach significantly reduced the number of insoluble particles of 2 μm and 5 μm. This reduction can be attributed to the fully enclosed mixing and dissolving process, which minimizes contamination risk. Additionally, the absence of a puncture step eliminates the introduction of particles associated with steel needles and rubber plug punctures. In summary, the powder–liquid DCB method effectively maintains the lowest possible level of insoluble particles in the final infusion.

### 4.4. Multidimensional Operational and Economic Evaluation for Clinical Decision-Support

To provide a practical decision-support framework, we have incorporated a preliminary, semi-quantitative multidimensional evaluation comparing PFI, PIVAS, and DCB. Based on institutional operational records and related administrative data, this theoretical comparison delineates the inherent clinical and economic trade-offs among the three models (Table 7).

As detailed in Table 7, the initial procurement premium of the DCB configuration (28.66 vs. 13.77 RMB/dose for conventional powder) is substantially offset by the complete elimination of secondary consumable costs, which otherwise add approximately 5 RMB and 8–10 RMB per dose to PFI and PIVAS, respectively. Operationally, centralized PIVAS introduces nearly 5 h of cumulative logistical latency (43-min dispatch plus 246-min transit), inherently exacerbating the risk of temperature-induced degradation. Conversely, the closed-system DCB bypasses these systemic delays, enabling total bedside admixture in approximately 22.5 s. By decoupling the preparation phase from prolonged delivery timelines, the ready-to-use DCB format translates preserved physicochemical stability into minimized medication waste, effectively justifying its higher baseline cost through integrated workflow and material efficiency.

## 5. Conclusions

Prolonged and continuous intravenous infusions of meropenem present a significant physicochemical liability, as hydrolytic degradation accelerates under room temperature and higher concentrations, rapidly driving Impurity A beyond pharmacopoeial limits. This study demonstrates that traditional admixture workflows and extended storage introduce unacceptable operational latencies that exacerbate this degradative process. By contrast, the deployment of powder–liquid DCBs effectively mitigates these risks through immediate, point-of-care compounding. The DCB system circumvents prolonged logistical delays, maintains structural stability of the active pharmaceutical ingredient, and significantly reduces insoluble particulate contamination. Consequently, for critical care scenarios such as septic shock—where therapeutic timeliness and formulation safety are paramount—DCBs offer a streamlined and prophylactic administration strategy. Future multidimensional health economic evaluations are warranted to optimize the cost–benefit ratio of these immediate-use admixture systems across diverse institutional settings.

## Figures and Tables

**Table 1:** Different sample configuration environments and storage conditions for meropenem injection at varying concentrations and environments.

Storage Environment	Norm	Configuration Environment	Samples	Serial Number
Refrigeration (2~8 °C)	0.5 g	sickroom	Meropenem for Injection (Meiping)	5-MW-C
	0.5 g	sickroom	Meropenem for Injection (Zhuojie)	5-ZW-C
	0.5 g	sickroom	Meropenem Sodium/Sodium Chloride for Injection	5-HW-C
	0.5 g	PIVAS	Meropenem for Injection (Meiping)	5-MP-C
	0.5 g	PIVAS	Meropenem for Injection (Zhuojie)	5-ZP-C
	1.0 g	sickroom	Meropenem for Injection (Meiping)	10-MW-C
	1.0 g	sickroom	Meropenem for Injection (Zhuojie)	10-ZW-C
	1.0 g	sickroom	Meropenem Sodium/Sodium Chloride for Injection	10-HW-C
	1.0 g	PIVAS	Meropenem for Injection (Meiping)	10-MP-C
	1.0 g	PIVAS	Meropenem for Injection (Zhuojie)	10-ZP-C
	2.0 g	sickroom	Meropenem for Injection (Meiping)	20-MW-C
	2.0 g	sickroom	Meropenem for Injection (Zhuojie)	20-ZW-C
	2.0 g	sickroom	Meropenem Sodium/Sodium Chloride for Injection	20-HW-C
	2.0 g	PIVAS	Meropenem for Injection (Meiping)	20-MP-C
	2.0 g	PIVAS	Meropenem for Injection (Zhuojie)	20-ZP-C
Room temperature (25 ± 5 °C)	0.5 g	sickroom	Meropenem for Injection (Meiping)	5-MW-R
	0.5 g	sickroom	Meropenem for Injection (Zhuojie)	5-ZW-R
	0.5 g	sickroom	Meropenem Sodium/Sodium Chloride for Injection	5-HW-R
	0.5 g	PIVAS	Meropenem for Injection (Meiping)	5-MP-R
	0.5 g	PIVAS	Meropenem for Injection (Zhuojie)	5-ZP-R
	1.0 g	sickroom	Meropenem for Injection (Meiping)	10-MW-R
	1.0 g	sickroom	Meropenem for Injection (Zhuojie)	10-ZW-R
	1.0 g	sickroom	Meropenem Sodium/Sodium Chloride for Injection	10-HW-R
	1.0 g	PIVAS	Meropenem for Injection (Meiping)	10-MP-R
	1.0 g	PIVAS	Meropenem for Injection (Zhuojie)	10-ZP-R
	2.0 g	sickroom	Meropenem for Injection (Meiping)	20-MW-R
	2.0 g	sickroom	Meropenem for Injection (Zhuojie)	20-ZW-R
	2.0 g	sickroom	Meropenem Sodium/Sodium Chloride for Injection	20-HW-R
	2.0 g	PIVAS	Meropenem for Injection (Meiping)	20-MP-R
	2.0 g	PIVAS	Meropenem for Injection (Zhuojie)	20-ZP-R
High temperature (40 ± 2 °C)	0.5 g	sickroom	Meropenem for Injection (Meiping)	5-MW-H
	0.5 g	sickroom	Meropenem for Injection (Zhuojie)	5-ZW-H
	0.5 g	sickroom	Meropenem Sodium/Sodium Chloride for Injection	5-HW-H
	0.5 g	PIVAS	Meropenem for Injection (Meiping)	5-MP-H
	0.5 g	PIVAS	Meropenem for Injection (Zhuojie)	5-ZP-H
	1.0 g	sickroom	Meropenem for Injection (Meiping)	10-MW-H
	1.0 g	sickroom	Meropenem for Injection (Zhuojie)	10-ZW-H
	1.0 g	sickroom	Meropenem Sodium/Sodium Chloride for Injection	10-HW-H
	1.0 g	PIVAS	Meropenem for Injection (Meiping)	10-MP-H
	1.0 g	PIVAS	Meropenem for Injection (Zhuojie)	10-ZP-H
	2.0 g	sickroom	Meropenem for Injection (Meiping)	20-MW-H
	2.0 g	sickroom	Meropenem for Injection (Zhuojie)	20-ZW-H
	2.0 g	sickroom	Meropenem Sodium/Sodium Chloride for Injection	20-HW-H
	2.0 g	PIVAS	Meropenem for Injection (Meiping)	20-MP-H
	2.0 g	PIVAS	Meropenem for Injection (Zhuojie)	20-ZP-H

**Table 2:** Insoluble particle count for each group at 0 h (*n* = 8).

Experimental Group/Particle Size	2 μm	5 μm	10 μm	25 μm
5-MW-C	606.5 ^a^	71.4 ^a^	0.5	0.4
5-MP-C	215.5 ^b^	46 ^b^	0.6	0.3
5-ZW-C	555.9 ^a^	83 ^a^	0.5	0.2
5-ZP-C	226.3 ^b^	48.5 ^b^	0.7	0.3
5-HW-C	33.6 ^c^	8.8 ^c^	0.5	0.2
5-MW-R	495.1 ^a^	68.5 ^c^	1.2	0.6
5-MP-R	268.1 ^c^	35.5 ^b^	0.2	0.1
5-ZW-R	391.8 ^b^	100.8 ^c^	0.6	0.3
5-ZP-R	218.3 ^c^	59.5 ^b^	0.5	0.3
5-HW-R	31.8 ^d^	5.7 ^a^	0	0
5-MW-H	521 ^a^	114.7 ^a^	0.3	0.3
5-MP-H	193.1 ^b^	46.8 ^b^	0.2	0.1
5-ZW-H	510 ^a^	109 ^a^	0.7	0.2
5-ZP-H	208.3 ^b^	42.2 ^b^	0.3	0.3
5-HW-H	33.8 ^c^	7.5 ^c^	0.5	0.2
10-MW-C	684.5 ^a^	96.4 ^b^	0.6	0.2
10-MP-C	221.8 ^b^	64.6 ^c^	0.6	0.3
10-ZW-C	743.2 ^a^	120.2 ^a^	0.6	0.3
10-ZP-C	205.7 ^b^	40.4 ^d^	0.6	0.2
10-HW-C	41.3 ^c^	7.6 ^e^	0.5	0.2
10-MW-R	698.7 ^a^	88.8 ^b^	0.4	0.2
10-MP-R	230.7 ^b^	64.3 ^c^	0.4	0.3
10-ZW-R	673.3 ^a^	112.2 ^a^	0.7	0.3
10-ZP-R	205.5 ^b^	40.5 ^d^	0.7	0.2
10-HW-R	34.8 ^c^	9.2 ^e^	0.5	0.2
10-MW-H	716.8 ^a^	63.4 ^b^	0.5	0.2
10-MP-H	30.6 ^b^	32 ^c^	0.2	0.3
10-ZW-H	621.5 ^a^	87 ^a^	0.8	0.1
10-ZP-H	186.5 ^b^	34 ^c^	0.5	0.3
10-HW-H	36 ^c^	9.8 ^d^	0.4	0
20-MW-C	1062.8 ^a^	115.8 ^b^	0.5	0.1
20-MP-C	231.9 ^b^	52.8 ^c^	0.4	0.1
20-ZW-C	1135.9 ^a^	183.4 ^a^	0.8	0.2
20-ZP-C	232.8 ^b^	46.5 ^c^	0.3	0.2
20-HW-C	170.2 ^b^	53.6 ^c^	2	0.1
20-MW-R	1288.3 ^a^	137.8 ^a^	0.6	0.3
20-MP-R	278.2 ^c^	46.8 ^b^	0.3	0.2
20-ZW-R	1068.9 ^b^	128.5 ^a^	0.8	0.2
20-ZP-R	317.8 ^c^	42.3 ^b^	0.3	0.1
20-HW-R	134.4 ^c^	37.1 ^b^	0.5	0.2
20-MW-H	495.1 ^b^	68.5 ^b^	1.2	0.6
20-MP-H	268.1 ^c,d^	35.5 ^c^	0.2	0.1
20-ZW-H	115.2 ^a^	168.9 ^a^	0.6	1
20-ZP-H	334.8 ^b,c^	80.5 ^b^	0.4	0.3
20-HW-H	112.8 ^e^	19.8 ^c^	0.3	0.1

Values with the same superscript letter are not significantly different (*p* ≥ 0.05), while those with different letters are significantly different (*p* < 0.05).

**Table 3:** Initial (0 h) concentrations, ranges, and RSDs of meropenem prepared by traditional powder-vial and dual-chamber bag methods at different dosages.

Experimental Group	Theoretical Concentration Values at the Initial Time Point (0 h)	Mean Concentration	Concentration Range		RSD
	(mg/mL)	(mg/mL)	Maximum Concentration (mg/mL)	Minimum Concentration (mg/mL)	
0.5 g/100 mL Meropenem was infused with traditional powder needles (*n* = 48)	500	500.1	538.3	448.6	3.33%
0.5 g/100 mL Meropenem finished infusion in powder and liquid dual-chamber bag (*n* = 24)	500	536.7	549.3	518.3	1.73%
1.0 g/100 mL Meropenem was infused with traditional powder needles (*n* = 48)	1000	997.1	1092.8	909.5	3.58%
1.0 g/100 mL Meropenem finished infusion in powder and liquid dual-chamber bag (*n* = 24)	1000	1068.9	1088.8	1039.3	1.29%
2.0 g/100 mL Meropenem was infused with traditional powder needles (*n* = 48)	2000	1975.1	2234.2	1846.5	4.36%
2.0 g/100 mL Meropenem finished infusion in powder and liquid dual-chamber bag (*n* = 24)	2000	2165.0	2230.5	2112.4	1.40%

**Table 4:** Absolute percentage of meropenem over 12 h under different storage conditions.

Conditions of Investigation		Meropenem Percentage (%, x ± s), Standard: 90.0~110.0%							
	Experimental Group (*n* = 8)	Initial Content	0 h	2 h	4 h	6 h	8 h	12 h	Inspection Results
Refrigeration (2~8 °C)	5-MW-C	997.8 ± 2.49	100	99.7 ± 0.6	99.5 ± 0.7	98.8 ± 0.7	98.7 ± 0.5	99.0 ± 1.1	There was no significant decrease in the content of each group under refrigeration conditions, and all groups met the pharmacopeial standard at 12 h.
	5-MP-C	977.4 ± 3.5	100	100.2 ± 0.6	99.6 ± 0.9	98.8 ± 1.3	98.6 ± 1.2	98.5 ± 1.1	
	5-ZW-C	1013.8 ± 2.8	100	99.9 ± 0.7	99.9 ± 0.9	99.4 ± 0.7	99.8 ± 0.6	99.2 ± 0.8	
	5-ZP-C	1005.7 ± 1.6	100	99.7 ± 0.6	99.8 ± 0.8	99.9 ± 0.9	99.9 ± 0.9	99.3 ± 1.0	
	5-HW-C	1091.6 ± 0.5	100	100.7 ± 0.6	100.5 ± 0.8	101.2 ± 2.5	98.3 ± 0.3	98.9 ± 0.9	
	10-MW-C	1005.0 ± 3.5	100	100.0 ± 0.2	99.7 ± 0.4	99.1 ± 0.6	98.7 ± 1.1	98.1 ± 1.1	
	10-MP-C	978.0 ± 3.6	100	100.1 ± 0.8	99.5 ± 0.7	99.4 ± 0.8	98.9 ± 0.7	98.9 ± 1.6	
	10-ZW-C	985.8 ± 3.5	100	99.9 ± 0.2	99.8 ± 0.4	100.9 ± 4.4	100.7 ± 4.3	100.1 ± 4.3	
	10-ZP-C	1003.5 ± 2.7	100	99.9 ± 0.2	99.8 ± 0.3	97.4 ± 4.6	96.4 ± 5.3	96.5 ± 6.1	
	10-HW-C	1080.39 ± 0.83	100	100.4 ± 1.2	99.9 ± 1.3	100.8 ± 1.0	100.4 ± 1.3	98.4 ± 1.1	
	20-MW-C	948.4 ± 1.3	100	99.6 ± 0.8	98.9 ± 2.0	99.1 ± 1.4	97.9 ± 2.0	98.5 ± 1.5	
	20-MP-C	985.4 ± 4.3	100	99.9 ± 0.8	98.9 ± 1.1	99.3 ± 1.3	99.4 ± 1.1	97.5 ± 1.1	
	20-ZW-C	1007.7 ± 4.5	100	100.0 ± 0.3	99.5 ± 0.7	99.0 ± 0.7	99.1 ± 0.9	98.7 ± 0.4	
	20-ZP-C	980.0 ± 2.4	100	99.7 ± 0.4	99.2 ± 0.5	98.9 ± 1.0	98.8 ± 0.8	98.7 ± 1.2	
	20-HW-C	1086.38 ± 1.60	100	100.2 ± 0.9	100.1 ± 0.9	99.5 ± 1.2	99.3 ± 1.2	98.7 ± 1.0	
Room temperature (25 ± 5 °C)	5-MW-R	996.2 ± 2.8	100	99.2 ± 1.2	98.9 ± 2.4	96.9 ± 1.7	97.1 ± 1.6	96.3 ± 1.0	At room temperature, the content with the extension of time continued to decline slightly. The 12 h high concentration of powder and liquid double-chamber bag group content does not meet the “Chinese Pharmacopoeia” minimum; the other groups of indicators are still in line with the “Chinese Pharmacopoeia” standards
	5-MP-R	996.9 ± 3.2	100	99.4 ± 2.4	99.3 ± 1.8	97.3 ± 1.2	97.1 ± 1.5	96.3 ± 0.8	
	5-ZW-R	1024.1 ± 2.7	100	98.7 ± 1.4	97.7 ± 1.9	97.6 ± 1.7	97.2 ± 1.5	96.1 ± 1.4	
	5-ZP-R	1002.4 ± 2.9	100	99.1 ± 0.4	98.0 ± 0.9	97.6 ± 0.9	97.3 ± 0.5	96.6 ± 0.8	
	5-HW-R	1054.2 ± 1.2	100	99.4 ± 1.7	99.5 ± 1.7	99.1 ± 1.9	97.3 ± 2.0	96.0 ± 1.3	
	10-MW-R	1057.7 ± 1.6	100	100.5 ± 1.4	99.2 ± 1.0	97.6 ± 0.9	96.4 ± 1.1	94.9 ± 1.8	
	10-MP-R	1003.3 ± 1.7	100	99.4 ± 0.6	99.6 ± 0.5	98.1 ± 1.3	97.2 ± 1.1	94.6 ± 1.8	
	10-ZW-R	1026.1 ± 2.7	100	98.6 ± 0.6	98.5 ± 1.1	97.9 ± 0.6	96.8 ± 1.3	94.3 ± 1.0	
	10-ZP-R	984.7 ± 2.8	100	99.0 ± 1.1	98.3 ± 1.0	97.6 ± 1.2	96.7 ± 1.4	94.4 ± 0.8	
	10-HW-R	1052.9 ± 0.7	100	99.8 ± 1.0	99.7 ± 1.1	99.0 ± 0.8	97.4 ± 1.0	94.7 ± 0.9	
	20-MW-R	952.4 ± 1.4	100	99.6 ± 1.5	98.4 ± 1.9	96.5 ± 2.3	95.0 ± 1.0	92.9 ± 0.9	
	20-MP-R	950.6 ± 2.6	100	98.4 ± 0.9	98.9 ± 0.7	97.4 ± 3.7	95.0 ± 4.0	92.8 ± 2.0	
	20-ZW-R	1043.4 ± 2.6	100	99.4 ± 1.2	98.2 ± 0.8	97.6 ± 0.9	96.9 ± 0.7	91.4 ± 1.0	
	20-ZP-R	952.8 ± 2.8	100	99.2 ± 0.5	98.3 ± 0.5	97.9 ± 0.5	97.0 ± 0.6	91.8 ± 1.3	
	20-HW-R	1079.4 ± 1.44	100	99.4 ± 0.8	97.5 ± 0.9	95.7 ± 1.8	93.2 ± 1.6	87.7 ± 1.7	
High temperature (40 ± 2 °C)	5-MW-H	994.8 ± 4.2	100	98.2 ± 0.4	95.0 ± 1.1	89.8 ± 1.9	87.5 ± 1.9	83.2 ± 2.3	Under high temperature conditions, the 6 h medium and low concentration groups did not meet the minimum requirements of the Pharmacopoeia; the 4 h high concentration groups did not meet the minimum requirements of the pharmacopoeia
	5-MP-H	985.5 ± 4.5	100	97.7 ± 0.9	94.6 ± 1.4	89.7 ± 3.0	87.9 ± 2.2	83.6 ± 3.8	
	5-ZW-H	1005.1 ± 2.3	100	97.8 ± 0.7	95.3 ± 1.2	90.0 ± 0.9	87.9 ± 0.8	82.6 ± 2.0	
	5-ZP-H	996.8 ± 2.2	100	97.5 ± 1.2	94.4 ± 1.1	89.8 ± 1.6	88.4 ± 1.7	83.11 ± 2.0	
	5-HW-H	1074.6 ± 1.1	100	98.2 ± 0.4	95.3 ± 0.7	90.8 ± 0.8	87.9 ± 1.1	83.5 ± 1.1	
	10-MW-H	972.7 ± 3.0	100	96.2 ± 1.8	92.3 ± 1.4	88.8 ± 1.3	85.5 ± 1.5	79.9 ± 4.6	
	10-MP-H	967.3 ± 4.6	100	96.2 ± 1.2	92.3 ± 1.6	88.6 ± 1.3	85.1 ± 3.7	79.6 ± 1.4	
	10-ZW-H	982.6 ± 3.7	100	96.8 ± 1.2	92.2 ± 1.4	88.4 ± 1.0	85.64 ± 2.0	79.8 ± 1.8	
	10-ZP-H	998.1 ± 3.0	100	96.4 ± 1.7	91.9 ± 1.0	88.6 ± 2.1	85.4 ± 1.9	79.7 ± 1.4	
	10-HW-H	1073.3 ± 0.4	100	96.8 ± 0.8	92.5 ± 1.3	88.7 ± 1.3	85.1 ± 0.7	79.2 ± 0.7	
	20-MW-H	1023.1 ± 5.2	100	95.8 ± 0.7	90.0 ± 1.2	85.8 ± 2.2	84.2 ± 3.0	75.0 ± 1.6	
	20-MP-H	1002.4 ± 1.5	100	95.1 ± 0.9	89.6 ± 1.5	85.6 ± 1.7	82.3 ± 1.5	75.8 ± 1.4	
	20-ZW-H	1020.2 ± 5.3	100	95.0 ± 1.3	88.6 ± 1.5	85.8 ± 1.8	83.3 ± 1.3	74.9 ± 1.2	
	20-ZP-H	984.2 ± 3.3	100	93.5 ± 1.2	88.8 ± 1.4	85.5 ± 1.5	83.6 ± 1.3	73.9 ± 1.5	
	20-HW-H	1081.74 ± 1.24	100	95.0 ± 1.4	89.9 ± 1.5	84.8 ± 1.4	81.6 ± 1.2	74.4 ± 1.4	

**Table 5:** Proportion of impurity A content over 12 h under different storage conditions.

Time	Impurity A Content (%, x ± s), Standard not More than 0.5						
	Contaminants A	0 h	2 h	4 h	6 h	8 h	12 h
Refrigeration (2~8 °C)	5-MW-C	0.12 ± 0.02	0.38 ± 0.05	0.42 ± 0.01	0.67 ± 0.04 ①	0.83 ± 0.07 ①	0.98 ± 0.05 ①
	5-MP-C	0.16 ± 0.03	0.32 ± 0.05	0.42 ± 0.01	0.67 ± 0.06 ①	0.83 ± 0.02 ①	1.01 ± 0.03 ①
	5-ZW-C	0.13 ± 0.02	0.29 ± 0.03	0.46 ± 0.01	0.61 ± 0.03 ①	0.84 ± 0.02 ①	0.97 ± 0.05 ①
	5-ZP-C	0.14 ± 0.04	0.31 ± 0.01	0.47 ± 0.01	0.68 ± 0.01 ①	0.79 ± 0.01 ①	1.05 ± 0.03 ①
	5-HW-C	0.17 ± 0.03	0.26 ± 0.04	0.34 ± 0.03	0.66 ± 0.04 ①	0.90 ± 0.08 ①	0.96 ± 0.03 ①
	10-MW-C	0.10 ± 0.01	0.29 ± 0.04	0.51 ± 0.02 ①	0.66 ± 0.02 ①	0.83 ± 0.03 ①	0.97 ± 0.07 ①
	10-MP-C	0.09 ± 0.02	0.40 ± 0.03	0.52 ± 0.02 ①	0.67 ± 0.01 ①	0.81 ± 0.03 ①	1.00 ± 0.05 ①
	10-ZW-C	0.14 ± 0.04	0.29 ± 0.04	0.53 ± 0.02 ①	0.57 ± 0.01 ①	0.84 ± 0.04 ①	1.01 ± 0.05 ①
	10-ZP-C	0.13 ± 0.02	0.40 ± 0.03	0.52 ± 0.03 ①	0.57 ± 0.00 ①	0.83 ± 0.03 ①	0.97 ± 0.05 ①
	10-HW-C	0.15 ± 0.04	0.13 ± 0.04	0.37 ± 0.02	0.77 ± 0.07 ①	0.86 ± 0.02 ①	0.98 ± 0.06 ①
	20-MW-C	0.26 ± 0.12	0.51 ± 0.09 ①	0.67 ± 0.05 ①	0.72 ± 0.06 ①	0.77 ± 0.02 ①	0.93 ± 0.07 ①
	20-MP-C	0.24 ± 0.07	0.52 ± 0.03 ①	0.60 ± 0.02 ①	0.69 ± 0.05 ①	0.79 ± 0.02 ①	0.94 ± 0.04 ①
	20-ZW-C	0.23 ± 0.06	0.29 ± 0.03	0.51 ± 0.02 ①	0.65 ± 0.09 ①	0.88 ± 0.01 ①	0.99 ± 0.10 ①
	20-ZP-C	0.22 ± 0.07	0.31 ± 0.01	0.54 ± 0.08 ①	0.67 ± 0.08 ①	0.87 ± 0.09 ①	1.02 ± 0.17 ①
	20-HW-C	0.19 ± 0.21	0.42 ± 0.18	0.53 ± 0.12 ①	0.81 ± 0.12 ①	0.98 ± 0.05 ①	1.04 ± 0.09 ①
Room temperature (25 ± 5 °C)	5-MW-R	0.10 ± 0.02	0.72 ± 0.02 ①	1.64 ± 0.14 ①	1.70 ± 0.04 ①	2.09 ± 0.12 ①	3.13 ± 0.08 ①
	5-MP-R	0.16 ± 0.05	0.76 ± 0.01 ①	1.69 ± 0.08 ①	1.68 ± 0.06 ①	2.02 ± 0.06 ①	3.26 ± 0.09 ①
	5-ZW-R	0.11 ± 0.01	0.70 ± 0.03 ①	1.23 ± 0.12 ①	1.75 ± 0.08 ①	2.18 ± 0.17 ①	3.19 ± 0.16 ①
	5-ZP-R	0.14 ± 0.04	0.73 ± 0.04 ①	1.34 ± 0.09 ①	1.70 ± 0.10 ①	2.15 ± 0.20 ①	3.18 ± 0.10 ①
	5-HW-R	0.09 ± 0.01	0.65 ± 0.08 ①	1.20 ± 0.12 ①	1.61 ± 0.17 ①	2.47 ± 0.32 ①	3.21 ± 0.26 ①
	10-MW-R	0.19 ± 0.04	0.79 ± 0.06 ①	1.25 ± 0.08 ①	1.66 ± 0.13 ①	2.37 ± 0.05 ①	3.31 ± 0.08 ①
	10-MP-R	0.17 ± 0.07	0.78 ± 0.06 ①	1.24 ± 0.06 ①	1.68 ± 0.12 ①	2.34 ± 0.12 ①	3.39 ± 0.13 ①
	10-ZW-R	0.15 ± 0.05	0.70 ± 0.11 ①	1.32 ± 0.09 ①	1.71 ± 0.03 ①	2.29 ± 0.06 ①	3.29 ± 0.10 ①
	10-ZP-R	0.17 ± 0.03	0.73 ± 0.04 ①	1.27 ± 0.05 ①	1.67 ± 0.06 ①	2.21 ± 0.03 ①	3.32 ± 0.12 ①
	10-HW-R	0.15 ± 0.04	0.75 ± 0.14 ①	1.14 ± 0.14 ①	1.61 ± 0.12 ①	1.96 ± 0.11 ①	3.29 ± 0.16 ①
	20-MW-R	0.23 ± 0.05	0.80 ± 0.13 ①	1.14 ± 6.49 ①	1.72 ± 0.21 ①	2.19 ± 0.16 ①	3.37 ± 15.26 ①
	20-MP-R	0.25 ± 0.04	0.84 ± 0.10 ①	1.17 ± 5.86 ①	1.70 ± 0.34 ①	2.40 ± 0.19 ①	3.35 ± 11.36 ①
	20-ZW-R	0.22 ± 0.04	0.91 ± 0.04 ①	1.14 ± 0.10 ①	1.70 ± 0.08 ①	2.19 ± 0.06 ①	3.30 ± 0.21 ①
	20-ZP-R	0.25 ± 0.08	0.92 ± 0.06 ①	1.13 ± 0.06 ①	1.71 ± 0.04 ①	2.24 ± 0.10 ①	3.37 ± 0.06 ①
	20-HW-R	0.22 ± 0.04	0.90 ± 0.05 ①	1.27 ± 0.09 ①	1.71 ± 0.04 ①	2.30 ± 0.02 ①	3.92 ± 0.04 ①
High temperature (40 ± 2 °C)	5-MW-H	0.13 ± 0.02	1.57 ± 0.18 ①	3.55 ± 0.10 ①	5.76 ± 0.17 ①	7.86 ± 0.14 ①	9.61 ± 0.25 ①
	5-MP-H	0.13 ± 0.02	1.65 ± 0.13 ①	3.53 ± 0.08 ①	5.66 ± 0.11 ①	7.84 ± 0.25 ①	9.90 ± 0.53 ①
	5-ZW-H	0.29 ± 0.47	1.67 ± 0.08 ①	3.74 ± 0.22 ①	5.40 ± 0.27 ①	7.54 ± 0.15 ①	10.33 ± 0.77 ①
	5-ZP-H	0.16 ± 0.04	1.54 ± 0.18 ①	3.80 ± 0.11 ①	5.77 ± 0.22 ①	7.65 ± 0.25 ①	10.43 ± 0.35 ①
	5-HW-H	0.14 ± 0.16	1.70 ± 0.12 ①	3.72 ± 2.76 ①	5.63 ± 3.80 ①	7.61 ± 4.84 ①	9.94 ± 1.83 ①
	10-MW-H	0.10 ± 0.18	1.84 ± 0.09 ①	3.70 ± 0.03 ①	5.91 ± 0.02 ①	7.51 ± 3.50 ①	10.14 ± 0.93 ①
	10-MP-H	0.11 ± 0.06	1.83 ± 0.08 ①	3.73 ± 0.03 ①	5.92 ± 0.02 ①	7.49 ± 3.12 ①	10.11 ± 0.89 ①
	10-ZW-H	0.14 ± 0.39	1.92 ± 0.05 ①	3.72 ± 0.02 ①	6.05 ± 2.14 ①	7.54 ± 1.00 ①	10.23 ± 0.98 ①
	10-ZP-H	0.11 ± 0.25	1.97 ± 0.11 ①	3.73 ± 0.03 ①	6.04 ± 1.28 ①	7.43 ± 2.07 ①	10.22 ± 0.92 ①
	10-HW-H	0.16 ± 0.19	1.87 ± 0.04 ①	3.72 ± 0.02 ①	6.00 ± 0.79 ①	7.51 ± 1.71 ①	10.22 ± 1.57 ①
	20-MW-H	0.22 ± 0.02	2.14 ± 0.04 ①	3.86 ± 0.07 ①	6.66 ± 0.38 ①	7.55 ± 0.38 ①	10.87 ± 0.27 ①
	20-MP-H	0.15 ± 0.03	2.14 ± 0.09 ①	3.78 ± 0.03 ①	6.67 ± 0.56 ①	7.45 ± 0.43 ①	10.72 ± 0.37 ①
	20-ZW-H	0.24 ± 0.01	1.99 ± 0.18 ①	3.57 ± 0.19 ①	6.58 ± 0.24 ①	7.67 ± 0.67 ①	10.64 ± 0.58 ①
	20-ZP-H	0.23 ± 0.00	2.03 ± 0.15 ①	3.36 ± 0.05 ①	6.47 ± 0.35 ①	7.55 ± 0.26 ①	10.46 ± 0.45 ①
	20-HW-H	0.27 ± 0.21	1.96 ± 0.06 ①	3.78 ± 0.08 ①	6.29 ± 0.04 ①	7.78 ± 0.05 ①	10.93 ± 0.06 ①

① Indicates that the content of Meropenem does not comply with the scope of the Chinese Pharmacopoeia.

**Table 6:** Comparison of the present study with previously published stability investigations of meropenem.

Clinical Scenario and Delivery System	Evaluated Parameters (Quality Indicators)	Storage Conditions (Time and Temperature)	Key Findings	Core Limitations vs. Novelty of the Present Study	Refs.
Continuous/extended infusion using infusors and conventional infusion bags	Chemical stability (HPLC), pH, and visual inspection	4.5 °C, 24.5 °C, 32 °C (Up to 5–6 days for infusors; 1 day for bags)	Admixtures in infusion bags maintained chemical stability and physical compatibility for at least 24 h. In contrast, stability in infusors was compromised by high concentrations and elevated temperatures; a 24 h infusion of high-dose meropenem (6 g) was only feasible when strictly maintained at 4.5 °C.	Focused strictly on physical compatibility and generic drug content; lacked quantitative assessment of specific degradation impurities and insoluble particles.	[14]
Outpatient parenteral antibiotic therapy (OPAT) using portable elastomeric infusion devices	Physicochemical stability (HPLC drug content > 95%), physical degradation	2–8 °C, 25 °C, 32 °C (Up to 24 h)	Low-concentration solutions (2 mg/mL) maintained >95% drug content without physical degradation for 8 h at 32 °C. However, high-concentration solutions (25 mg/mL) failed to support prolonged infusion, with stability compromised beyond 8 h under refrigeration (2–8 °C) or beyond 4 h at elevated temperatures (25 °C and 32 °C).	Evaluated elastomeric devices under controlled conditions; omitted specific evaluation of particulate matter and strict pharmacopoeial impurity limits.	[15]
Automated dispensing cabinets and bedside preparation using addEASE^®^ connectors	Meropenem concentration recovery (>90% threshold via HPLC-UV and LC-MS/MS)	Room temperature (Up to 6 h)	Meropenem admixtures (2 g in 100 mL normal saline) prepared via dispensing connectors maintained an active ingredient recovery of >90% from baseline throughout a 6 h observation window at room temperature.	Limited observation window (6 h); assessed active ingredient recovery only, without addressing degradation byproducts or particulate safety.	[16]
Critical care and sepsis management using powder–liquid double-chamber bags (DCBs) vs. traditional powder-for-injection	Content uniformity (HPLC), specific Impurity A levels against pharmacopoeial limits, insoluble particle counts, pH, osmolality, and appearance	2–8 °C, 25 ± 5 °C, 40 ± 2 °C (Up to 12 h)	DCBs demonstrated superior content uniformity and significantly fewer insoluble particles than traditional formulations. Under refrigeration, meropenem content remained >95% with suppressed impurity formation for up to 12 h; however, Impurity A exceeded pharmacopoeial limits within 2 h at room and elevated temperatures.	Provides a multidimensional safety evaluation incorporating compendial Impurity A and particulate limits; features an innovative bedside volume adjustment for high-concentration critical care dosing.	Present study

**Table 7:** Multidimensional preliminary cost-effectiveness evaluation of PFI, PIVAS, and DCB configurations.

Evaluation Dimension	PFI (Ward-Based Preparation)	PIVAS	DCB
Drug Cost	Low: Volume-based procurement (VBP) domestic powder injection: 13.77 RMB/vial; original powder injection: 109.45 RMB/vial	Medium: VBP domestic/original powder injection + sterile solvent + basic PIVAS operational costs	High: 28.66 RMB/bag
Time-cost	High: Requires substantial manual preparation time exclusively by ward nurses	Medium: Exact per-bag preparation time is currently difficult to standardize; however, the process from packaging to dispatch requires approx. 43 min [29], and the transit from dispatch to clinical receipt averages 246 min	Low: Experimental activation takes 5–10 s/bag; the actual clinical admixture time is approximately 22.5 s/bag [30,31]
Medical Supplies	Present: Approx. 5 RMB/dose (includes basic preparation consumables such as syringes, sterile solvents, and disinfectants)	Approx. 8–10 RMB/dose (includes basic consumables, plus cleanroom-specific materials and specialized protective equipment for admixture)	Requires no additional preparation consumables; the system is directly activated via manual pressure
Stability-related Waste	Relatively High: Manual preparation at the ward is susceptible to administration delays, leading to potential stability degradation and subsequent medication waste	High: Advance batch preparation increases the risk of wastage due to unpredictable changes in clinical prescriptions; additionally, prolonged delivery procedures exacerbate stability-related losses	Low: Ready-to-use formulation eliminates the need for advance preparation and mitigates delivery delays, making stability-related waste highly controllable

## Data Availability

The original contributions presented in this study are included in the article. Further inquiries can be directed to the corresponding author.
